# Characteristics of the level of psychomotor abilities of female handball players

**DOI:** 10.1186/s13102-022-00475-5

**Published:** 2022-05-05

**Authors:** Maciej Śliż, Karolina H. Przednowek, Paweł Kapuściński, Bartosz Dziadek, Łukasz Godek, Krzysztof Warchoł, Janusz Zieliński, Krzysztof Przednowek

**Affiliations:** 1 Institute of Physical Culture Sciences, Medical College of Rzeszów University, Rzeszów University, Rzeszów, Poland; 2 Department of Sport Games, Józef Piłsudski University of Physical Education in Warsaw, Warsaw, Poland

**Keywords:** Reaction time, Movement time, Women, Team sport, Competition class, Body composition

## Abstract

**Background:**

Handball is a high-intensity game, during which players run, change directions with or without the ball, interact with the opponent and make different decisions in both offensive and defensive actions. Players‘ performance may depend on a number of factors, including explosive force, power, speed and agility. Players‘ results may be significantly influenced by their, psychomotor abilities. This article presents an analysis of selected psychomotor abilities of female handball players at different level of competition.

**Methods:**

Test2Drive computer tests were used. The following four tests were used to measure psychomotor abilities: the Simple Reaction Time Test (SIRT), the Choice Reaction Time Test (CHORT), the Hand-Eye Coordination Test and the Spatial Anticipation Test (SPANT). The study covered a group of 118 female handball players (average age 19.6 ± 3.16), playing in the PGNiG Polish Women’s Superliga, the Polish 1st Handball League and 2nd the Polish 2nd Handball League, in the playing positions: goalkeepers, centre players, pivot players and wing players. The study also included analyses of the players‘ body composition and aerobic capacity through the use of the multistage 20-m shuttle run test. In addition, the players‘ psychomotor abilities were analysed depending on the level of competition and playing position.

**Results:**

The analysis of the reaction time and movement time showed statistically significant differences between the results obtained by the female handball players on different levels of competition. Moreover the female players from the Polish Women’s Superliga exhibited the fastest reaction times according to the SIRT, the CHORT and the SPANT. Additionally, on the basis of the linear Pearson correlation coefficient, a statistically significant relationship was determined between the players‘ psychomotor abilities (movement time in the SIRT, the CHORT and the SPANT) and elements of their body composition or aerobic capacity.

**Conclusions:**

The analysis showed that the higher the level of competition (leagues), the shorter the female handball players‘ reaction times. The study also revealed that the players‘ body mass index and percentage of fat content reassociated with movement times, while their aerobic capacity (measured in the multistage 20-m shuttle run test) had a significant influence on their reaction times. This study shows that reaction time it’s one of ability which should be consider to develop in training of female handball players.

## Background

Handball is one of the most popular Olympic team games in the world. It is currently played by more than 19 million people and since 1972 has been an Olympic sport for men [1]. By contrast, handball became an Olympic sport for women in 1976 [2]. Over the past few years, the speed of both male and female handball players has increased significantly. The possibility of quickly resuming the game after a goal is scored by an opponent—a rule introduced in 2000 [3]—and tactical changes consisting in combining a position attack with a quick attack [4–7], have enabled game play to become more dynamic. Handball is characterised by high-intensity game, during which players run, change directions with or without the ball, interact with the opponent and make different decisions in both offensive and defensive actions [8–12].

The results achieved in today’s handball may depend on a number of factors [4]. Previous studies investigating the motor abilities of players have suggested that explosive force, power, speed and agility are important factors [12–16]. Players‘ results may also be significantly influenced by their coordination abilities [17], defined as a complex capacity correlated with speed, strength, endurance and mobility [18]. Moreover, players‘ coordinative capacities are part of the physical abilities. It is also a fundamental task in motor learning [19].

The specificity of each player‘s playing position can also be crucial to sporting achievement [20, 21]. Female players in the pivot position are characterised by their high level of divisibility of attention, those in the centre position by their spatial orientation and divisibility of attention and those in the wing position by their complex reaction time, frequency of movement and simple reaction time [22]. Michalsik, Aargaard and Madsen [23] have shown that wing players use more sprints and have fewer physical confrontations than back players and pivots. Female handball players playing in the Italian elite and sub-elite championships have similar linear anthropometry and body mass index (BMI) [24]. The different positions (wing, centre, pivot and goalkeeper) have different technical, tactical, physiological demands and anthropometric features [25]. In particular, wings and goalkeepers significantly differ in several anthropometric and body composition parameters. Milanese [24] has also found differences between wings and centre players (body high, body mass comp; right arm; mass of left leg), between pivots and goalkeepers (triceps skinfold; fat mass of right arm), and between backs and goalkeepers (BMI).

Previous studies have indicated that players‘ psychomotor abilities may also be crucial to the effectiveness of the game. Psychomotor abilities are key in open sports and can determine success or failure of the individual athlete or a team. The cognitive training consists of a variety of brain exercises designed to improve performance of psychomotor skills e.g. decision making, anticipation, fast response time [26]. Analysing statistics and recordings of F.C. Barcelona matches during the Champions League 2011–2012, Curiţianu et al. [27] have shown that that players‘ reaction speeds and use of appropriate defence systems have an impact on the number of effective quick attacks. The differences in time anticipation between Champions League and the PGNiG Superliga goalkeepers suggest that top-level goalkeepers wait until the last second before interfering, which makes them more effective and give the thrower an opportunity to change the target of the throw, which may translate into a lower effectiveness of intervention [7]. Similarly, analysing match statistics of 60 qualifying matches for the World Women‘s Handball Championship in 2003, Ohnjec et al. [28] have stated that the possibility of a fast break is determined by reaction speeds. In addition, a players‘ reaction speed combined with their speed and efficiency on the court during a counterattack gives a good chance of scoring a goal. Rata [29] has also observed the impact of speed and reaction time on the final result of a handball match. On the other hand, Bideau et al. [30] have demonstrated that professional goalkeepers‘ abilities to anticipate and reduce their reaction times during goal interventions influence their levels of effectiveness. Other studies have assessed selected psychomotor abilities, relating them to various aspects of matches [31–33] or training [34], using various tests [31, 34] and measurement systems [32, 33]. For instance, on the basis of the tests conducted, Cicma [34] has determined the reaction speeds of people practicing handball in relation to the training measures. Furthermore, taking into account the ages and the position of female junior players of the Serbian handball team, Marković et al. [31] have examined the simple visual reaction time and the variability of reaction time. On the basis of the laboratory tests and methods used, the authors determined the statistical significance of the results of the study groups. It was also noted that loads and complexity of the training process for example visual response time, adapted to the requirements of modern handball selection, requires more professional players to be tested. A similar conclusion was reached by Kajtana et al. [33], who using the CRD system, attained the simple reaction, simple visual orientation, simple selective response and complex visual orientation reaction times among 46 goalkeepers. Based on the obtained results and the level of advancement of the players (divided into two groups, successful and less successful), the authors indicated the need to compare goalkeepers with wing, centre and pivot players. The psychomotor abilities of 11 professional handball goalkeepers playing in the PGNiG Superliga in Poland have also been assessed by Krawczyk et al. [32] who used the Vienna Test System to determine and compare the players‘ reaction times, motor times, DT motor times and ZBA time anticipation with their defensive effectiveness during wing players‘ throws.

A player‘s reaction time has also been related to their playing position [21, 31], seniority and experience [33, 35] as well as competition class (leagues) and as already mentioned, motor abilities [16]. Psychomotor abilities, including reaction time, can play a key role and lead to victory [36–40]. Yüksel and Tunç [40] have indicated that the reaction times of the leading teams are shorter than their lower classified counterparts. This factor, in addition to technique and tactical preparation, has a major influence on a team‘s likelihood of achieving victory [40]. Similarly Mańkowska et al. [38] have shown that players‘ perceptive abilities combined with their abilities to predict movement, can facilitate success in team sports.

For this reason, research in to players‘ psychomotor abilities, the factors that determine their level and the impact of these abilities on sports results is justified and essential. In this context, in order to increase knowledge about the importance and role of psychomotor abilities in female handball, the authors assessed selected psychomotor abilities of professional female handball players from Poland, taking into account their competition class (league), playing position, body composition, BMI and aerobic capacity. In addition, authors investigated which of the above factors affects the average level of the considered abilities and which of them significantly differentiates the results obtained in computer psychomotor tests.

## Methods

### Characteristics of the study group

The study covered a group of 118 professional female handball players from different levels of competition (average age: 19.6 ± 3.16, training seniority: 7.69 ± 2.43). On the players are associated with the Polish Handball Federation and participate at various levels of women‘s hanball in Poland: the PGNiG Polish Women’s Superliga, the Polish 1st Handball League and the Polish 2st Handball League. The players are on leading teams and were given permission to participate in the research by the Polish Handball Federation. The study was performed with 17 female players from the Polish Women’s Superliga, 63 from the Polish 1st Handball League and 38 from the Polish 2nd Handball League. The all playing positions were taken into account: goalkeeper (GK) (20 players), centre player (CP) (52 players), pivot player (PP) (14 players) and wing player (WP) (32 players). Each player‘s height and mass and body composition (bio-electrical impedance analysis) were measured. In addition, their aerobic capacity was measured with the use of the multistage 20-m shuttle run test (20mSRT) [41, 42]. The characteristics of the test group are presented in Table 1.

**Table 1:** Sample characteristics

Variable	Total	Super league	I league	II league	*p*	e.s.
	N = 118	N = 17	N = 63	N = 38		
Age$^\diamond$	19.60±3.16	17.24±1.25	20.83±3.39	18.63±2.35	0.001*	0.21
Body mass (kg)$^\diamond$	65.79±8.03	67.45±5.78	67.47±7.09	62.27±9.29	0.001*	0.13
Body height (cm)$^\diamond$	170.84±6.23	173.00±6.76	172.29±6.25	167.47±4.49	0.001*	0.15
BMI$^\diamond$	22.46±2.38	22.56±1.80	22.62±2.18	22.16±2.90	0.213	0.03
FAT (%)$^\diamond$	26.66±5.31	27.65±4.39	27.56±4.95	24.75±5.85	0.018*	0.07
FFM (kg)$^\diamond$	47.72±3.94	48.65±3.31	48.21±3.38	46.48±4.78	0.005*	0.09
TBW (kg)$^\diamond$	34.57±2.93	35.19±2.29	34.84±2.55	33.86±3.63	0.025*	0.06
20mSRT (m)$^\diamond$	1695±317	1999±393	1744±248	1477±229	0.001*	0.28
Position$^\dag$						
CP	52 (44.07)	8 (47.06)	32 (50.79)	12 (31.58)		
GK	20 (16.95)	3 (17.65)	9 (14.29)	8 (21.05)	0.818	0.16
PP	14 (11.86)	1 (5.88)	8 (12.70)	5 (13.16)		
WP	32 (27.12)	5 (29.41)	14 (22.22)	13 (34.21)		

Data are expressed as: $\diamond$ mean ± standard deviation, $\dag$ n (%); * statistical significance

*e.s.* effect size, *20mSRT* — distance in 20-m shuttle run test

### Measurement of psychomotor abilities

**Fig. 1:**
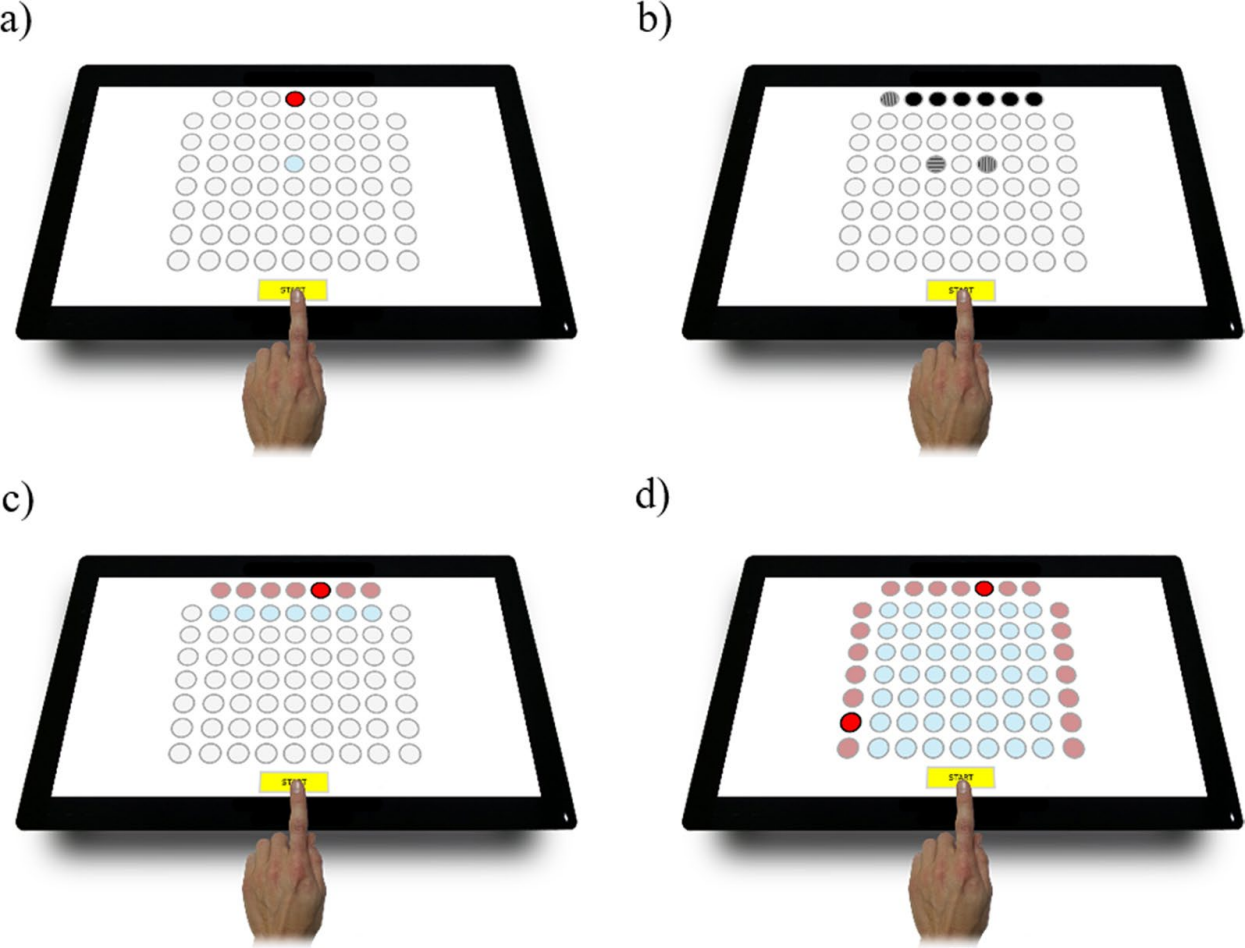
Reaction panel of the Test2Drive system; **a** SIRT, **b** CHORT, **c** HECOR, **d** SPANT

As the study method, Test2Drive (ALTA, Siemianowice Slaskie, Poland) psychometric computer tests were used (Fig. 1). The various studies were subjected by Test2Drive system. Tarnowski et al. [43] have reviewed and described the characteristics of these tests and have confirmed their theoretical validity. The Test2Drive system fulfils all the requirements of the Regulation of the Polish Minister of Health. The analyses of the female handball players were divided into 2 days. During the first day in the morning, the measurement consisted of four computer system tests, which were used to measure the players‘ psychomotor abilities. The measurements were taken in a standing position so that the participants could perform certain tasks freely. The screen was kept in a horizontal position during all four tests. Following the instructions, the exercise stage took place, during which the study participants could learn the protocol of stimuli presentation and giving responses. This stage was followed by the proper testing stage. The study participants had to react as quickly as possible to certain stimuli in all tests. In each type of test, the stimuli appeared at different intervals. On the second day, measurements were taken through the 20mSRT in order to estimate each player‘s level of fitness. Before undergoing the fitness test, all the participants warmed up on a handball court. Test2Drive was used to measure indicators of psychomotor abilities through the Simple Reaction Time Test (SIRT), the Choice Reaction Time Test (CHORT), the Hand-Eye Coordination Test (HECOR) and the Spatial Anticipation Test (SPANT). Reaction time (RT) and movement time (MT) were the main indicators in all the tests, while the percentage of correct responses was calculated and analysed in the CHORT and the SPANT.

## Figures

Information on the four tests is summarised below:

- SIRT: used to evaluate reaction speed and its stability. The stimuli-signalling field changed colour in the right moment of research time. Responding to the stimuli involved moving one‘s finger from the START field to the reaction time field marked in blue.
- CHORT: used to evaluate the speed and adequacy of a reaction in a complex situation. Horizontal (stimuli) benchmarks and vertical stimuli which required a reaction and a slant benchmark (neutral stimuli) that did not require a reaction, were displayed in the top signalling row. Responding to the stimuli involved moving one’s finger from the START field to one of the two reaction fields (vertical or horizontal stimulus field). During the neutral stimuli, the participant was asked to keep her finger on the START field.
- HECOR: used to evaluate hand-eye coordination. The test required careful observation of the board and providing a quick reaction to the red signalling field displayed. The test participant was asked to move her finger from the START field to the blue reaction field and then return her finger to the START field.
- SPANT: used to evaluate hand-eye coordination via complex spatial information. At the top, and on the left and right sides of the test board were signalling fields, two of which (on in a row and one in a column) turned red simultaneously. In response to the stimulus, the test participant was asked to indicate with her finger the field on the crossing of the lit row and column and then return her finger to the START field.

In all the tests the stimuli exposure time lasted 3 s, while the stimulation intervals lasted either 1 s, 1.5 s or 2 s. Each test lasted 3 min. RT and MT were measured for each test, while in the CHORT and the SPANT, the percentage of correct responses was also determined. In the SIRT, the HECOR and the SPANT, 20 stimuli were used, while in the CHORT, 24 cases were used.

### Statistical methods

Basic statistical measures (arithmetic mean, standard deviation and size of individual groups) were used in the study. For the dependent variables analysed, the normality of the distribution was examined (the Shapiro–Wilk test). Given that some of the variables lacked a normal distribution, non-parametric tests were used in further analyses. In order to identify the significance of the differences between the groups, the Kruskal–Wallis and U Mann–Whitney tests were used. The effect size was calculated using the following formulas [44]:

- for Kruskal–Wallis – $E_R^2=\dfrac{H(N+1)}{(N^2-1)}$,
- for U Mann–Whitney test – $r=\dfrac{Z}{\sqrt{N}}$,
- for chi-squared test – $\phi =\sqrt{\dfrac{x^2}{N}}$

where *H*—the Kruskal–Wallis H-test statistic, *N*—the total number of observations, $E_R^2$—epsilon-squared coefficient assumes the value from 0 to 1.00, *Z*—standardized value for the U-value, *r*—correlation coefficient where *r* assumes the value ranging from − 1.00 to 1.00. The analysis was performed with the GNU R software [45].

## Results

Table 2 presents the individual psychomotor abilities of the female handball players analysed, depending on the level of competition. The analysis revealed that the Superliga players had the lowest scores and thus higher RT, SIRT, CHORT and SPANT levels than 1st and 2nd League counterparts. In addition, in the case of the CHORT and the SPANT, this group of subjects was characterised by the highest percentages of correct answers (CHORT—95.29%; SPANT—91.76%). The situation differed in the case of MT: in this group of subjects (Superliga), the best results were recorded in the MT SIRT (216.29 ms), the MT CHORT (234.00 ms), the MT HECOR (259.71 ms) and the MT SPANT (277.18 ms). It should also be noted that the best RT values in the individual psychomotor tests were recorded among the group of women with the lowest overall fitness level, i.e. the 2nd League (RT SIRT—352.79 ms; RT CHORT—698.71 ms; RT HECOR—414.61 ms; RT SPANT—642.82 ms). In addition, a statistical relationship between the players‘ level of competition and psychomotor test results was noted for the MT SIRT and the MT HECOR and the RT CHORT.

**Table 2:** Characteristics of psychomotor abilities of female handball players

Variable	Total	Super league	1st league	2nd league	*p*	$\mathbf{E_R^2}$
	(N = 118)	(N = 17)	(N = 63)	(N = 38)		
Simple reaction time test (SIRT)						
RT (ms)	347.22 ± 36.26	333.88 ± 26.24	347.46 ± 37.34	352.79 ± 37.54	0.268	0.02
MT (ms)	198.44 ± 36.95	216.29 ± 32.71	201.54 ± 37.40	185.32 ± 34.23	0.017*	0.07
Choice reaction time test (CHORT)						
RT (ms)	676.58 ± 65.63	650.82 ± 45.07	670.17 ± 70.85	698.71 ± 58.77	0.014*	0.07
MT (ms)	217.41 ± 40.96	234.00 ± 34.60	218.94 ± 43.06	207.45 ± 38.05	0.092	0.04
C.R. (%)	93.89 ± 6.12	95.29 ± 3.24	92.95 ± 7.27	94.82 ± 4.71	0.419	0.01
Hand–eye coordination test (HECOR)						
RT (ms)	408.78 ± 45.52	406.29 ± 73.67	405.94 ± 39.08	414.61 ± 39.95	0.245	0.02
MT (ms)	240.50 ± 36.39	259.71 ± 27.95	241.73 ± 39.15	229.87 ± 31.52	0.017*	0.07
Spatial anticipation test (SPANT)						
RT (ms)	617.65 ± 102.60	603.82 ± 79.52	606.21 ± 103.69	642.82 ± 107.58	0.265	0.02
MT (ms)	266.63 ± 62.77	277.18 ± 46.70	264.68 ± 60.29	265.13 ± 73.26	0.552	0.01
C.R. (%)	90.34 ± 9.10	91.76 ± 7.28	90.87 ± 9.00	88.82 ± 9.96	0.487	0.01

*RT*—reaction time, *MT—*movement time, *C.R.*—correct responses, *p*—probability of testing, $E_R^2$—effect size

*Statistical significance

**Table 3:** Correlations between the selected of body composition and distance variables and psychomotor test results

Variable	Group	SIRT		CHORT		HECOR		SPANT	
		RT	MT	RT	MT	RT	MT	RT	MT
Age	Total	ns	ns	ns	ns	ns	ns	ns	− 0.20
	II	ns	ns	ns	ns	ns	ns	ns	− 0.33
Body height (cm)	Total	ns	ns	ns	ns	ns	ns	ns	ns
Body mass (kg)	Total	ns	0.26	ns	0.24	ns	ns	ns	0.22
	I	ns	ns	ns	ns	0.25	ns	ns	ns
	II	ns	0.33	ns	0.37	ns	ns	ns	0.34
BMI	Total	ns	0.20	ns	0.31	ns	ns	ns	0.24
	I	ns	ns	0.25	0.26	ns	ns	ns	ns
	II	ns	ns	ns	0.40	ns	ns	ns	0.37
FAT (%)	Total	ns	0.22	ns	0.24	ns	ns	ns	0.22
	II	ns	ns	ns	ns	ns	ns	ns	0.35
FFM (kg)	Total	ns	0.19	ns	ns	ns	ns	ns	ns
	I	ns	ns	ns	ns	0.35	ns	ns	ns
	II	ns	0.36	ns	0.36	ns	ns	ns	ns
TBW (kg)	Total	ns	0.18	ns	ns	ns	ns	ns	ns
	I	ns	ns	ns	ns	0.35	ns	ns	ns
	II	ns	0.35	ns	0.39	ns	ns	ns	ns
20mSRT (m)	Total	ns	ns	− 0.32	ns	ns	ns	− 0.22	ns
	Super	ns	ns	− 0.72	ns	ns	ns	ns	ns
	I	ns	ns	ns	ns	− 0.28	ns	ns	ns
	II	ns	ns	ns	ns	ns	ns	ns	− 0.37

*RT*—reaction time, *MT*—movement time, *20mSRT*—distance in 20-m shuttle run test, *ns*—not significant, *Total*—all female handball players, *Super*—Super league, *I*—1st league, *II*—2nd league

In the next step, the correlations among the various variables (age, body height (cm), body mass (kg), BMI, FAT%, FFM (kg), TBW (kg), 20mSRT) and psychomotor test results were analysed according to the players‘ fitness levels (Table 3). No relevant dependencies were found for the age and height variables in any of the study groups in the case of the SIRT, the CHORT and the HECOR. Only during the last attempt to anticipate the analysis showed a significantly negative age correlation with the MT SPANT (total and 2nd League). In addition, body mass, BMI and FAT% were significantly positively correlated with the MT SIRT, the CHORT and the SPANT. It should be added that this phenomenon was observed for the entire study group (for the 2nd League in the MT CHORT and the SPANT). The analyses also revealed a significant link between distance and the RT CHORT (total and Superliga), the RT HECOR (1st League), the RT SPANT (total) and the MT SPANT (2nd League). In each of these cases, the correlation took a negative direction.

Table 4 presents each player’s response time, depending on their playing position. The analysis revealed that the CPs scored the weakest RT values in each psychomotor test. In addition, the best RT SIRT values were recorded among the WPs (353.25 ms) and the best RT CHORT and RT HECOR values were obtained by GKs (RT CHORT—703.95 ms and RT HECOR—419.80 ms). However, in the case of the RT SPANT, the worst results were observed for PPs (640.86 ms). At the same time, the results did not show any statistical significance.

**Table 4:** Basic statistics of psychomotor abilities of handball players—Position

Variable	CP	GK	PP	WP	*p*	$\mathbf{E_R^2}$
	(N = 52)	(N = 20)	(N = 14)	(N = 32)		
SIRT						
RT (ms)	339.08 ± 35.38	354.65 ± 32.61	353.07 ± 39.01	353.25 ± 37.57	0.176	0.04
MT (ms)	200.29 ± 34.49	207.45 ± 37.45	200.29 ± 34.41	189.00 ± 41.10	0.272	0.03
CHORT						
RT (ms)	667.06 ± 62.23	703.95 ± 67.51	673.00 ± 67.63	676.50 ± 67.33	0.155	0.04
MT (ms)	219.79 ± 40.03	228.40 ± 43.12	219.36 ± 44.69	205.81 ± 38.61	0.264	0.03
C.R. (%)	94.67 ± 5.68	91.90 ± 7.46	96.00 ± 3.84	92.94 ± 6.44	0.117	0.05
HECOR						
RT (ms)	406.23 ± 49.84	419.80 ± 35.10	407.14 ± 39.71	406.75 ± 47.16	0.408	0.02
MT (ms)	242.42 ± 36.42	245.35 ± 33.82	243.14 ± 38.27	233.19 ± 37.70	0.653	0.01
SPANT						
RT (ms)	604.94 ± 103.53	622.10 ± 96.90	640.86 ± 108.22	625.38 ± 104.10	0.594	0.02
MT (ms)	265.58 ± 64.53	263.00 ± 60.97	282.93 ± 67.52	263.47 ± 60.75	0.857	0.01
C.R. (%)	91.15 ± 8.20	90.00 ± 10.00	86.79 ± 12.34	90.78 ± 8.34	0.706	0.01

*RT*—reaction time, *MT*—movement time, *C.R.*—correct responses, *p*—probability of testing, $E_R^2$—effect size

*Statistical significance

## Discussion

This study aimed to assess selected psychomotor abilities (simple reaction time, choice reaction time, hand-eye coordination and anticipation) depending on female handball players‘ league, playing position and selected body composition parameters. The research has clearly shown that players in the PGNiG Polish Women’s Superliga have the greatest psychomotor abilities, with the exception of hand-eye coordination. This conclusion is congruent with another study regarding a group of male professional handball players [46]. The results of this study also correspond to findings from research into other sports. For instance, similar dependencies were found among fencers [47, 48]. Furthermore, football players with high fitness levels manifested much shorter reaction times after a 12-min run than players with low fitness levels [35]. Moreover, Kida et al. [36] found that among baseball players, tennis players and non-athletes, the response times of those with higher fitness levels were lower than those of players with lower fitness levels. Studies on the impact of significant physical effort on choice reaction time confirm these relationships [49], as this time improves together with increased energy expenditure, which is related to physical effort. The present study also revealed a statistically significant relationship between handball players‘ level of competition and choice reaction times.

In addition to the level of competition, the playing position of the player is an important factor influencing the reaction time. Przednowek et al. [46] have observed lover SIRT, HECOR and SPANT results, among centre players than goalkeepers, wing players and pivot players. It should be noted that these studies were carried out on a group of men, while in the present study, the same phenomenon was observed with a group of women. Nevertheless, this results did not prove to be statistically significant. Similarly, Polluveer [50] has observed this phenomenon among female volleyball players. Moreover, the simple and complex reaction time and anticipation time results were not found to differ significantly among players in different positions on the court.

This study also sought to identify the relationship between female handball players‘ psychomotor abilities and body composition. A positive correlation was found between the players‘ BMI and psychomotor test results. For instance, it was observed those players with either a higher body mass, BMI or FAT, FFM and TBW content manifested higher simple and choice reaction time values. Most research in this field has been carried out on groups of non-athletic people [51–54]. Studies by Moradi and Esmaeilzadeh on groups of children do not indicate any significant relationship between clinical reaction time and individual obesity indicators, such as BMI and body fat [54–56]. A significant positive correlation between BMI and visual and auditory reaction times has been observed by Nikam and Gadkari [57] and has since been confirmed by Skurvydas [51]. People with a higher BMI react significantly slower than those with a lower BMI. Deore et al. [58] have observed that girls who are overweight have higher auditory and visual reaction times than girls with an average body mass. The authors additionally observed a statistically significant relationship for visual reaction time. Few studies have addressed the relationship between psychomotor abilities and body composition among athletes. Studies on a group of tennis players have shown that visual reaction time negatively correlates with BMI. However, it should be noted that the correlation of visual reaction time and BMI has been performed across the study population (tennis players and healthy controls) [59]. Similar phenomena have been observed among runners [60]. Meanwhile, Mohammad et al. [61] have not confirmed this phenomenon. The results of studies performed on a group of male football players show that there is no relationship between body fat and reaction time [61].

The present study has also shown that there is a significant correlation between aerobic capacity and certain psychomotor abilities. In each of the cases analysed, a negative relationship was recorded. It was observed that with increased distance travelled during the 20mSRT, lower response time values were recorded. This phenomenon was seen for the CHORT (Superliga), the SPANT (total and 2nd League) and the HECOR (1st League). Such findings are congruent with those of Maghsoudipour et al. [62] regarding female track and field athletes.

The limitations of the study were related to the research group. Only the data of female handball players without the control group was analyzed in the study. A control group of non-athletes helped to describe in detail the influence of handball training on the development of psychomotor abilities. In the future the studies can be extended to the examine the relationship between psychomotor abilities and effectiveness of playing during the match of female handball.

## Conclusions

The results of the study revealed a relationship between the BMI of female handball players and the percentage of body fat on the one hand, and their movement time as measured by SIRT, CHORT and SPANT on the other. At the same time, it was found that the aerobic fitness of women in handball is related to CHORT and SPANT. In addition, the results showed that the players’ psychomotor skills were dependent on their handball competitive class. The best reaction times were recorded by players from the Superliga (SIRT, CHORT and SPANT), while the shortest movement times were achieved by players with the lowest level of competition (II league). This supports the conclusion that reaction time it’s one of ability which should be consider to develop in training of female handball players. The conducted research has shown that it is necessary to further monitor handball players’ reaction time, which will allow for detailed training related to working on a faster reaction in defense as well as in position and quick attack in handball.

## Data Availability

The data used to support the findings of this study are available from the corresponding author upon request.The data sets generated and analysed during the current study are available.
